# Distal femoral arthropometry in Nigerians and its correlation to total knee replacement implants

**DOI:** 10.4314/gmj.v55i4.3

**Published:** 2021-12

**Authors:** Olasode I Akinmokun, Nwachukwu N Ibeabuchi, Sekinat A Adejumobi, Abisola S Ajayi, Oyinlola O Thomas

**Affiliations:** 1 Department of Surgery, College of Medicine, University of Lagos, Idi-Araba, and Department of Orthopaedics, Lagos University Teaching Hospital, Idi-Araba, Nigeria; 2 Department of Anatomy, College of Medicine, University of Lagos, Idi-Araba, Nigeria; 3 Medical student, College of Medicine, University of Lagos, Idi-Araba, Nigeria

**Keywords:** total knee replacement, pre-operative, anthropometry, knee prosthesis, femoral component

## Abstract

**Introduction:**

Total Knee replacement (TKR) is performed to relieve pain and restore both the mechanical axis and joint line whenever indicated. Most of prostheses being used for TKR were manufactured using dimensions from Caucasians' measurements. This study documented the dimensions of distal femora of Nigerians and correlated the dimensions with different TKR prostheses.

**Materials and methods:**

Fifty-six matured femora were measured. Dimensions of distal femora from other regions were retrieved from published articles. The dimensions of TKR prosthesis were extracted from product monographs. Analyses were done with Microsoft excel 2010 (Microsoft Corporation, Redmond, Washington, United States) and STATA version 13 (StataCorp, Texas. USA). Statistical significance was set at p ≤ 0.05

**Results:**

The average Mediolateral dimension was 79.3 ± 4.4 mm. The anterioposterior dimensions of the medial and lateral condyles were 63.7 ± 3.6 mm and 64.9 ± 3.3 mm respectively. There were no significant differences between the left and right distal femur. The average aspect ratio calculated was1.23 ± 0.05. There was a mismatch of aspect ratio of the distal femora and those of the prostheses. Equations that can be useful both clinically and in forensic medicine were generated.

**Conclusion:**

This study has provided measurements that can be considered when the designing of a suitable femoral component of total knee prosthesis for Nigerians. This study also provided equations that can be used to estimate the dimensions of the medial and the lateral condyles and femoral length from parts of distal femur in forensic medicine.

**Funding:**

Self-funded

## Introduction

Total knee replacement (TKR) surgery has become a worldwide procedure, performed on patients with chronic moderate to severe pain that is refractory to all non-surgical modalities.^1^,^2^ This procedure restores both the mechanical axis and joint line of the affected limb.^3^ Indications for Total Knee Replacement includes osteoarthritis , rheumatoid arthritis, spondyloarthropathies, ankylosing spondylitis, psoriatic arthropathy, and posttraumatic arthritis secondary to intra-articular fracture.^1^,^2^ During this procedure, total knee Prostheses (comprising femoral and tibial components) are used to replace the degenerated ends of the articulating bones which are cut off.

Most of these prostheses were manufactured using dimensions from Caucasian femora and tibiae osteometric measurements. Physical morphologies differ among racial groups, and so also bony morphologies. Anthropometric and osteometric studies from different racial groups and regions of the world had shown differences in the dimensions of the distal femoral bones around the world. 4–10 Implants developed and manufactured based on the dimensions of a racial group might not fit another racial group. Thilak et al^4^, in their study amongst Indian patients, documented that all the implants used in their study showed some amount of mismatch with patients' anatomic dimensions with resultant poorer knee society scoring system in those with larger mediolateral mismatch after 2 years. It is, therefore, important for implants to be manufactured based on the dimension of the distal femoral bones of the populace of the region where it would be used.

This study was aimed to document the dimensions of distal femura of Nigerians and to compare these dimensions with the documented dimensions from other regions of the world. It also correlated the dimensions of the distal femura with different femoral components of total knee prostheses that are available. Statistical relationships between parts of distal femora were conducted through Pearson's chi square and regression analysis to devise equations that can be used for estimation of different parts of the bone, either for clinical use or in forensic medicine.

## Methods

Ethical approval was obtained from the Lagos University Teaching Hospital Hospitals' Ethics and Research Committee (HREC)-ADM/DCST/HREC/APP/2389 before the commencement of the study.

**Figure 1:**
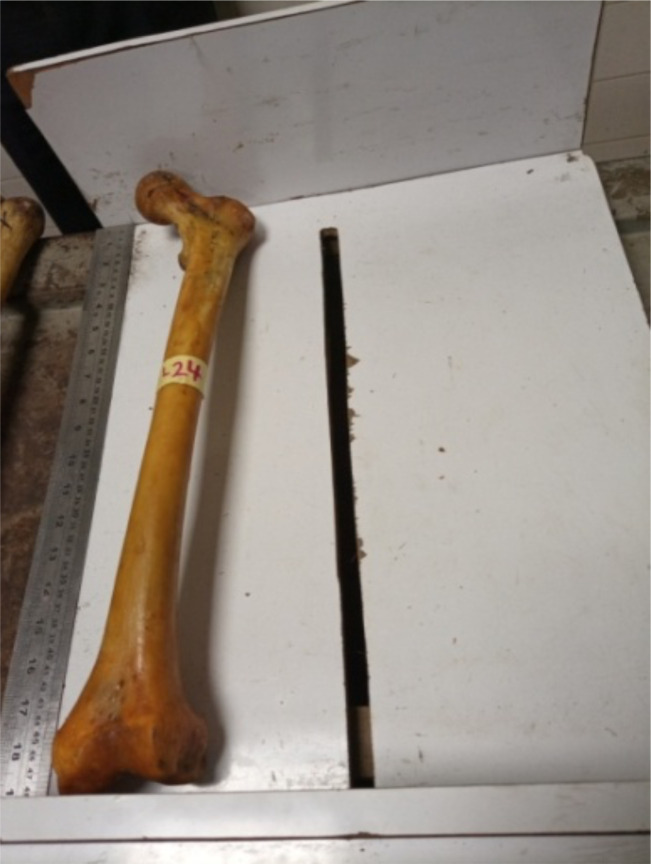
Maximum femoral length (MFL)

**Figure 2:**
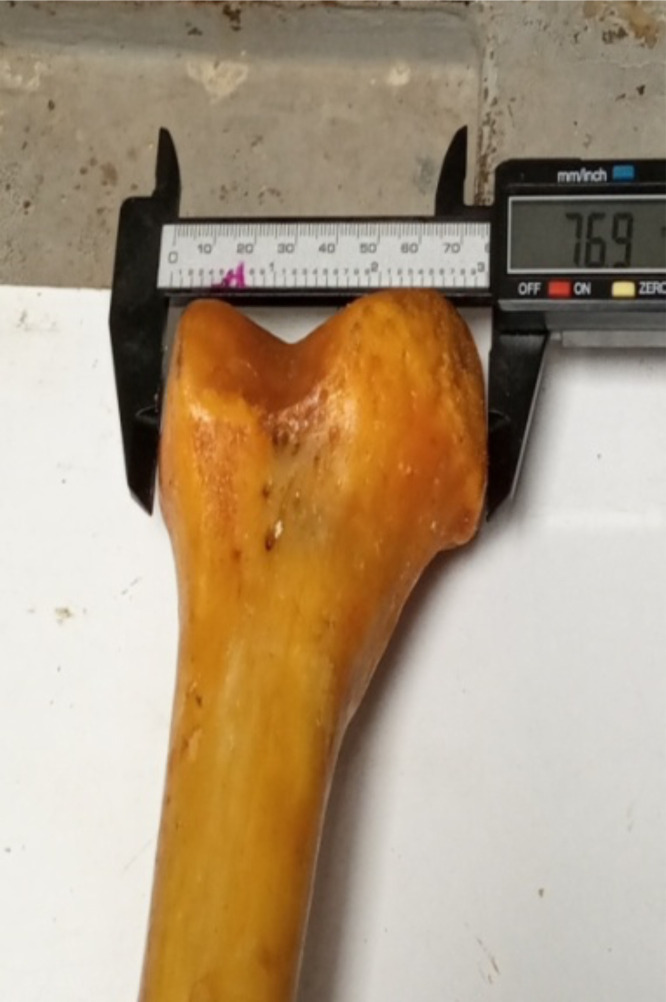
Mediolateral dimension (MLD)

**Figure 3:**
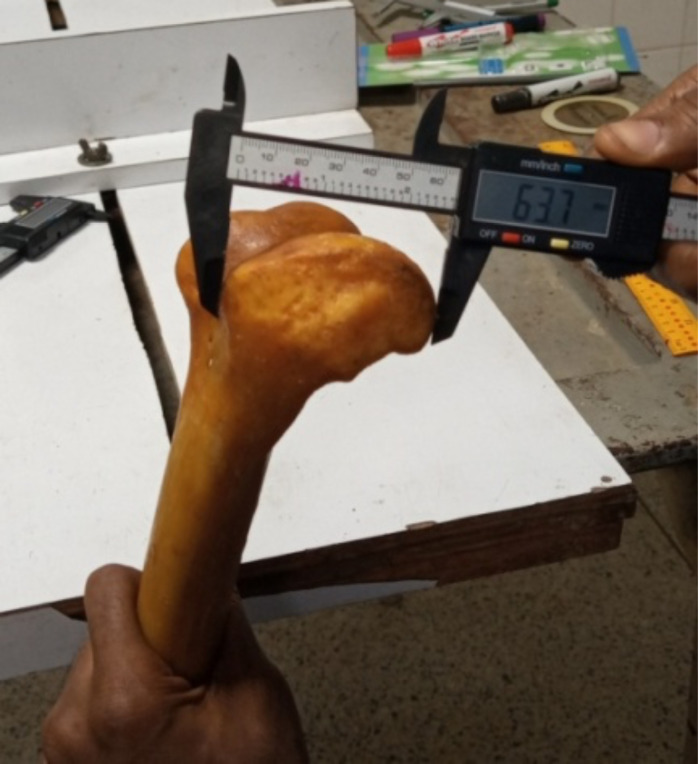
Anterior-Posterior Diameter of Medial Condyle (APDMC)

The dry femoral bones were retrieved and measured within the Department of Anatomy, College of Medicine, University of Lagos. A total of 61 femoral bones were retrieved but five were excluded because they were deformed and distorted. Fifty-six non-sexed, nonpaired, adult dry femoral bones were measured. They were sorted into laterality resulting in 27 right and 29 left femoral bones. Each of the bone was given an alphanumerical identity and labeled. The femoral bones with right laterality were labeled from R1 to R27 while those of the left laterality were labeled L1 to L29. Measurements were done with the osteometric board and digital vernier calipers. All measurements were done twice, and the average of the values was documented and used for the analysis. The dimensions measured were:

- Maximum Femur length (MFL): the distance from the highest border of femoral head to the lowest border of the medial condyle. (Figure 1)
- Femoral Trochanteric length (TL): the distance from the tip of the greater trochanter of femur to the lowest border of the lateral condyle.
- Medio-lateral dimension (MLD) of the femoral condyles – the widest dimension between the medial and lateral margins of the femoral condyles. (Figure 2)
- Anterior-Posterior Diameter of Medial Condyle (APDMC) - the maximum anterior-posterior dimension of the medial condyle of the distal femur. (Figure 3)
- Transverse Diameter of Medial Condyle (TDMC) - the maximum medial to lateral dimension of the medial condyle of the distal femur.
- Anterior-Posterior Distance of Lateral Condyle (APDLC) - the maximum anterior-posterior dimension of the lateral condyle of the distal femur.
- Transverse Diameter of Lateral Condyle (TDLC) - the maximum medial to lateral dimension of the lateral condyle of the distal femur.
- Intercondylar notch (IN) – the width of intercondylar notch

The aspect ratios of the femora were calculated by dividing the Medio-lateral dimension (MLD) of the femoral condyles with Anterior-Posterior Dimension of Lateral Condyle (APDLC). The dimensions of the femoral components of the total knee prostheses implants that were used in this study were obtained from their product monographs. Analyses were done with Microsoft Excel 2010 (Microsoft Corporation, Washington, United States) and STATA version 13 (StataCorp, Texas. USA). Mean and standard deviation were documented. Pearson's Chi square and regression analyses were done for associations. Statistical significance was set at p ≤ 0.05

## Results

Fifty- six (56) made up of 27 bones with right laterality and 29 bones of left laterality were measured. The average MFL for both sides were 47.9 ±2.1 cm which ranged from 42.1 cm to 51.4cm. The average distal femoral MLD dimension was 79.3 ± 4.4cm (ranged from 65.9cm to 90.4cm) and the APDMC and APDLC were 63.7 ± 3.6cm (ranged from 56.4cm to 71.8cm) and 64.9 ± 3.3cm (ranged from 57.5cm to 71.6cm) respectively. Table 1 shows a summary of the measured parameters. There were no significant differences between the dimensions of the left-sided and right-sided distal femur specimens.

**Table 1:** Measured parameters from the distal femora

Parameter	Right	Left	Average value	Minimum	Maximum	p value
**MFL (cm)**	47.8 ± 2.2	48.0 ± 2.0	47.9 ± 2.1	42.1	51.4	0.8569
**TL (cm)**	46.0 ± 2.1	45.5 ± 1.9	45.8 ± 2.0	40.2	50.0	0.7701
**MLD (mm)**	81.3 ± 3.5	77.7 ± 4.4	79.3 ± 4.4	65.9	90.4	0.3607
**APDMC (mm)**	63.8 ± 3.7	63.7 ± 3.6	63.7 ± 3.6	56.4	71.8	0.7869
**TDMC (mm)**	24.6 ± 2.2	23.6 ± 2.8	24.1 ± 2.6	18.8	28.8	0.7782
**APDLC (mm)**	65.2 ± 3.2	64.7 ± 4.2	64.9 ± 3.3	57.5	71.6	0.6169
**TDLC (mm)**	26.7 ± 2.5	26.3 ± 2.6	26.5 ± 2.6	20.2	30.7	0.6727
**IN (mm)**	18.2 ± 2.5	19.8 ± 2.3	19.0 ± 2.4	13.4	23.7	0.9160

Table 2 shows a comparison of the measured dimensions with measurements from other populations. The average aspect ratio of the distal femora was 1.23 ± 0.05 and ranged from 1.06 to 1.31

**Table 2:** Distal femora parameters and the aspect ratios from different regions of the world

Author	Ethnicity /Racial group	MLD (mm)	APDLC (mm)	APDMC (mm)	Aspect ratio
**Current study**	Nigerians	79.3 ± 4.4	64.9 ± 3.3	63.7 ± 3.6	1.23
**Lakati et al** 5	Kenyans	68.4 ± 5.19	61.2 ± 4.17	58.0	1.12
**Terzidis *et al*** 10	Greek	83.9±6.3	58.5±4.0	58.7±4.1	1.43
**Shah *et al*** 8	Indian	71.5 ± 2.5 (M) 65.1 ± 3.1(F) 68.3 ± 3.9(C)	65.6 ± 3.8(M) 59.8 ± 4.3(F) 62.7 ± 4.8(C)		1.09 ± 0.04(M) 1.09 ± 0.05(F) 1.09 ± 0.05(C)
**Kwak *et al*** 7	Korean	70.2±5.5	43.9±3.8		1.3
**Loures et al** 6	Brazilians	70.6 ±6.1	64.0±6.3		1.1
**Schmidt et al** 10	Asians Caucasians	70.5± 5.8 73.6± 6.2	50.7± 3.9 52.7± 3.6	51.1±3.9 52.8± 3.6	1.39 1.39

**Figure 4:**
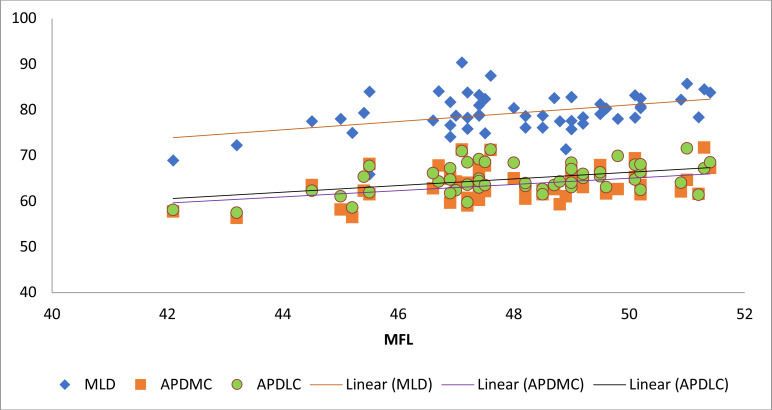
Scatter plot showing the relationship between MFL and MLD, APDMC, APDLC

**Figure 5:**
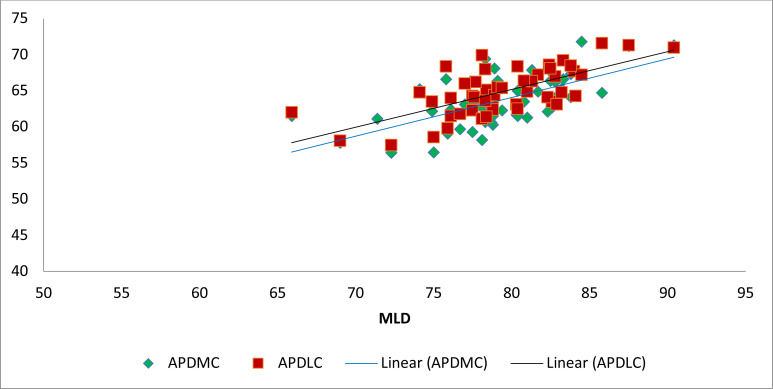
Scatter plots showing the relationship between MLD and APDMC, APDLC

Regression analysis revealed significant statistical relationships between the lengths of the femur and parts that made up the distal femur. (Figure 4 & 5) There were also significant statistical relationships between different parts that made up the distal femur. Equations were generated from the regression analyses Table 3.

**Table 3:** Statistical relationship between measured parameters

PARAMETER	MFL	TL	MLD
**TL**	p = 0.0001 MFL = 3.1 + 0.98*TL		
**MLD**	p = 0.0018 MFL = 32.72 + 1.6*MLD	P = 0.0006 TL = 29.9 + 2*MLD	
**APDMC**	p = 0.0047 MFL = 34.3 + 2.2*APDMC	p = 0.0038 APDMC = 29.777 + 0.074*TL	p = 0.0001 APDMC = 21.2 + 0.54*MLD
**TDMC**	p = 0.3687 Not significant	P =0.2327 Not significant	p = 0.0005 TDMC = 2.5 + 0.27*MLD
**APDLC**	p = 0.0008 MFL = 29.82 + 2.8*APDLC	p = 0.0028 APDLC = 33.2 + 0.069*TL	p = 0.0001 APDLC = 23.3 + 0.52*MLD
**TDLC**	P = 0.2408 Not significant	P = 0.2042 Not significant	p = 0.0001 TDLC = 0.53 + 0.33*MLD

**Figure 6:**
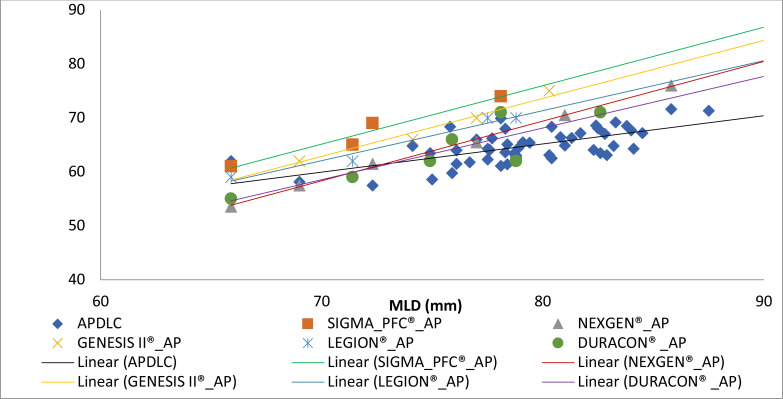
The dimensions of the distal femora and the femoral components

The scatter plot of the MLD and APDLC of the specimens and the corresponding dimensions of the femoral components of the total knee replacement available were plotted. (Figure 6) The measured dimensions of the distal femoral bones were smaller than those of the implants. All the trend lines in the plot showed positive slopes. However, the trend lines of the femoral prostheses had steep slopes compare to the trend line of the femoral condyles with gentle positive slope.

**Figure 7:**
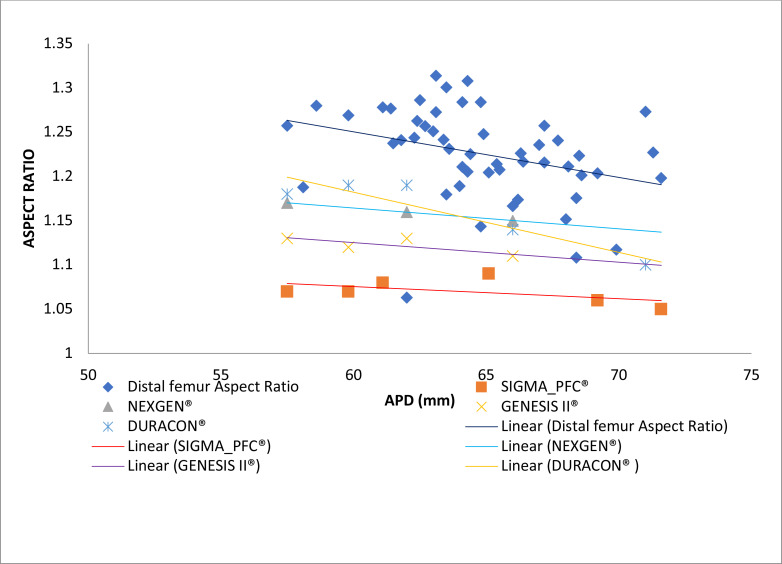
Aspect ratios of the distal femora and femoral prostheses

The aspect ratios of the distal femora were plotted against the aspect ratios of the femoral prostheses. (Figure 7) there was no overlapping between the aspect ratios of the distal femur and those of the femoral prostheses. The trend line of the distal femur showed a gentle negative slope. Only the trend line of the DuraconR prosthesis showed a similar trend while others were without or almost without gradients.

## Discussion

The average dimensions of the distal femur in Nigerians were documented in this study. The average distal femoral MLD dimension was 79.3 ± 4.4 mm and the anteroposterior dimensions of the medial APDMC and lateral APDLC condyles were 63.7 ± 3.6 mm and 64.9 ± 3.3 mm respectively. The dimensions of the MLD and APD were also similar to the values documented by Thilak et al^4^ in male Indians but larger than the values documented in Kenyans^5^, Brazilians^6^, Asians 7, 8, 9 It is however smaller than the values documented among Greek.^10^ This has further confirmed previous documentation on variations in dimensions of bones from different racial groups and regions of the world. This study has therefore provided data that can be used by implant manufacturing companies. Using dimensions from other racial groups may not produce prostheses that would fit our population appropriately.

The scatter plot of the MLD and APDLC of the distal femora specimens and the corresponding dimensions of the femoral components of the total knee replacement revealed that measured values of the distal femora were smaller and mostly concentrated within a particular range. The trend lines of the dimensions of the prostheses were steeper compared with the gentle slope of the distal femora. This implies that the increment in dimensions of the femoral prostheses from a lower size to the next available size was larger (both in APDLC and MLD dimensions) than the natural increment observed in the distal femora. This could be a source of mismatch during total knee replacement. It is therefore important for implants manufacturers to review available implants to produce implants that will fit our population.

The aspect ratios of the distal femora plotted against the aspect ratios of the femoral prostheses showed no overlapping between the aspect ratios. The aspect ratios of the Caucasians were used to produce these prostheses and may account for the difference. More so, the trend line of the distal femur showed a gentle negative slope. Almost all the trend lines for the aspect ratios of the prostheses were without or almost without a gradient except the trend line of the DuraconR prosthesis with a similar trend like the aspect ratios of the distal femora.

The gentle negative slope of the distal femora implied aspect ratios that decreased as the dimensions were getting larger. It must be stated that one of the draw-back of this study was the sample size, which was quite small.

The study generated equations that can be used clinically as part of pre-operative planning for total knee replacement or in forensic medicine for identification purposes. Some of the equations included

Equations to estimate the femoral condyles
  - APDMC = 21.2 + 0.54*MLD
  - TDMC = 2.45+ 0.27*MLD
  - APDLC = 23.3 + 0.52*MLD
  - TDLC = 0.53 + 0.33*MLD

The dimensions of the femoral condyles can be estimated from the mediolateral dimension (MLD). MLD can be measured clinically

The APDMC and APDLC dimensions can also be estimated from TL, which can also be measured clinically, from the tip of the greater trochanter to the lateral knee joint. These equations are:

- APDMC = 29.8 + 0.074*TL
- APDLC = 33.2 + 0.069*TL

b. Equations to estimate the maximum femoral length (in forensic medicine)
  - MFL = 32.72 + 0.16*MLD
  - MFL = 34.3 + 0.22*APDMC
  - MFL = 29.82 + 0.28*APDLC
  - MFL = 3.1 + 0.98*TL

## Conclusion

This study has documented average dimensions of the distal femora in Nigerians. It also provided data that can be considered when designing a suitable femoral component of a total knee prosthesis for Nigerians. This study provided equations that can be used to estimate the dimensions of the medial and the lateral condyles and femoral length in forensic medicine.
